# Enhanced Neuroactivation during Working Memory Task in Postmenopausal Women Receiving Hormone Therapy: A Coordinate-Based Meta-Analysis

**DOI:** 10.3389/fnhum.2015.00035

**Published:** 2015-02-11

**Authors:** Ke Li, Xiaoyan Huang, Yingping Han, Jun Zhang, Yuhan Lai, Li Yuan, Jiaojiao Lu, Dong Zeng

**Affiliations:** ^1^Key Laboratory for NeuroInformation of Ministry of Education, University of Electronic Science and Technology of China, Chengdu, China; ^2^School of Life Science and Technology, University of Electronic Science and Technology of China, Chengdu, China

**Keywords:** hormone therapy, ALE meta-analysis, functional magnetic resonance imaging, postmenopause, working memory, neural activation

## Abstract

**Background and Aim:** Hormone therapy (HT) has long been thought beneficial for controlling menopausal symptoms and human cognition. Studies have suggested that HT has a positive association with working memory, but no consistent relationship between HT and neural activity has been shown in any cognitive domain. The purpose of this meta-analysis was to assess the convergence of findings from published randomized control trials studies that examined brain activation changes in postmenopausal women.

**Methods:** A systematic search for fMRI studies of neural responses during working memory tasks in postmenopausal women was performed. Studies were excluded if they were not treatment studies and did not contain placebo or blank controls. For the purpose of the meta-analysis, 8 studies were identified, with 103 postmenopausal women taking HT and 109 controls.

**Results:** Compared with controls, postmenopausal women who took HT increased activation in the left frontal lobe, including superior frontal gyrus (BA 8), right middle frontal gyrus (BA 9), anterior lobe, paracentral lobule (BA 7), limbic lobe, and anterior cingulate (BA 32). Additionally, decreased activation is noted in the right limbic lobe, including parahippocampal gyrus (BA 28), left parietal lobe, and superior parietal lobule (BA 7). All regions were significant at *p* ≤ 0.05 with correction for multiple comparisons.

**Conclusion:** Hormone treatment is associated with BOLD signal activation in key anatomical areas during fMRI working memory tasks in healthy hormone-treated postmenopausal women. A positive correlation between activation and task performance suggests that hormone use may benefit working memory.

## Introduction

The influence of hormone treatment (HT) on brain and cognition in postmenopausal women has been a controversial topic. Cross-sectional studies and meta-analyses have promoted the idea that HT might enhance verbal memory performance and decrease the risk for developing dementia in postmenopausal women (Barrett-Connor and Stuenkel, 2001; LeBlanc et al., 2001; Maki, 2006). Studies indicated that HT users were better protected against cognitive decline and dementia and may have better performance during working memory tasks than non-users (Hogervorst et al., 2009; Davis et al., 2013). Similarly, a follow-up study of women who began hormone use around menopause detected lower cognitive impairment (Bagger et al., 2005). In a memory study, early initiators of HT performed better than late initiators in working memory tasks, including attention, concentration, and mental status (MacLennan et al., 2006). These studies thus suggest that HT may have beneficial effects on the central nervous system. However, other studies pointed out that HT has no detrimental effect on cognitive performance in early postmenopausal women (Davison et al., 2013). Later HTs have no preventive effect on AD and MCI in postmenopausal women (Maki, 2005; Sherwin, 2005). While most women receiving estrogen plus progestin did not experience clinically relevant adverse effects on cognition compared with placebo, a small increased risk of clinically meaningful cognitive decline occurred in the estrogen plus progestin group (Espeland et al., 2010). There are no adverse or beneficial effects of HT on cognitive function in younger postmenopausal women (Brown, 2013). Similarly, large controlled studies have suggested that HT has no beneficial effects on maintaining cognition (Henderson et al., 2000; Mulnard et al., 2000; Wang et al., 2000; Resnick et al., 2006). Moreover, data from the only large randomized controlled trial published to date, the Women’s Health Initiative Memory Study, did not confirm these observations and have even suggested an increase in dementia risk for women using hormonal replacement therapy compared to controls (Ryan et al., 2008). Based on currently available data, routine therapeutic use of estrogens in women with AD is not justified (Markou et al., 2005) and should not be used for dementia prevention (NIA, 2004). Thus the effect of HT on working memory remains unclear based on the results of studies involving direct manipulations of estradiol level.

Inconsistencies in estradiol effects are also observed in structural and functional brain imaging studies. Increases or decreases in activation are often not accompanied by changes in task performance, leading Maki and Resnick (Maki et al., 2011) suggest that brain imaging may be more sensitive to effects of estradiol or other hormones than task performance. Evidence from animal models has found estrogen receptors in numerous sites throughout the brain including the hippocampus, amygdala, hypothalamus, brainstem, and cerebral cortex suggesting that estrogen therapy may impact cognitive functioning through potential affects on these brain areas (Brake et al., 2001). It was also shown that estrogens enhance basal forebrain, hippocampus, and cortex cholinergic activity by influencing the synthetic enzyme for acetylcholine as well as by increasing the number of cholinergic neurons (Smith et al., 2001). Further, estrogen has been suggested to modulate various neurotransmitters (Moses et al., 2000; Smith et al., 2001), increase cerebral blood flow (Greene, 2000; Slopien et al., 2003), regulate the formation of synapses, affect neuronal survival (Nilsen et al., 2000; Gleason et al., 2005), influence the expression of APOE, and provide neuroprotective effects, all of which may either directly or indirectly impact cognitive functioning (Slopien et al., 2003). The fMRI results demonstrate that HT, relative to placebo, increased the response of the striatum and ventromedial prefrontal cortex, two areas that have been shown to be, respectively, involved during reward anticipation and at the time of reward delivery. Using both visual and verbal working memory tasks, one fMRI study found increased activation bilaterally in the superior frontal gyrus and in the inferior parietal lobule in subjects who were receiving high-dose conjugated equine estrogen compared with controls, while decreases in inferior parietal, which activation for storage of non-verbal material (Shaywitz et al., 1999), have also been reported. Areas of activation were deemed significantly different in the prior findings.

Although prior studies have examined the effects of HT on working memory task performance in healthy postmenopause women, to our knowledge, no brain imaging meta-analysis examined the brain activation differences in postmenopausal women. Our study assessed the convergence of findings from published randomized control trials studies that examined brain activation changes in postmenopausal women. A meta-analysis employing the activation likelihood estimate (ALE) method was performed to locate anatomical regions with significant activation differences. The ALE method permits the creation of three-dimensional (3D) probability maps that show the brain regions that are most likely to demonstrate morphometric differences between HT and controls. The frontal lobes are of paramount significance in determining emotions and judgments related to sympathy, which is defined as the ability to perform daily activities, personality manifestations, and decisions (Badre et al., 2009; Tiemeier et al., 2010). Base on the exiting studies (Shaywitz et al., 1999; Greene, 2000; Moses et al., 2000; Nilsen et al., 2000; Brake et al., 2001; Smith et al., 2001; Slopien et al., 2003; Gleason et al., 2005), we hypothesize that postmenopausal women who took HT would show increased activation in the frontal lobe, including temporal lobe and the anterior cingulate, brain regions known to be involved in cognition and working memory.

## Methods

### Data sources and inclusion criteria

**Figure 1:**
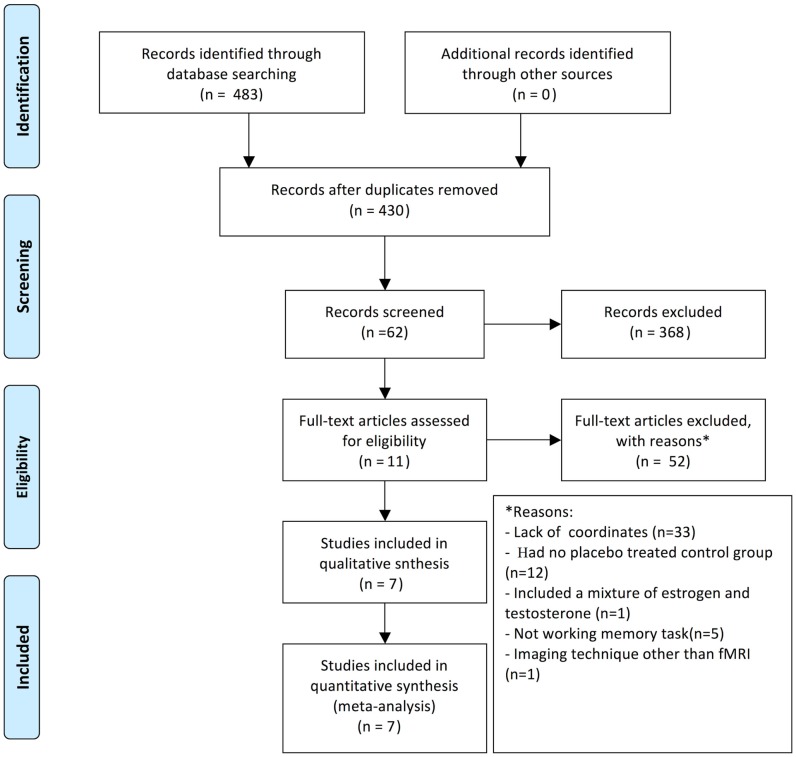
**Diagram of studies included in the present meta-analysis**.

A systematic search strategy was performed to identify the relevant studies. We searched PubMed, MEDLINE, and EMBASE from 1993 to 2014 for all relevant published observational studies and clinical trials. The following key terms were employed: “HT,” “HRT,” “HT,” “ERT,” “functional MRI or fMRI,” “cognitive dysfunction,” “postmenopausal,” “brain activation,” and “working memory.” The reference lists of these articles were searched to obtain additional relevant studies. All identified articles were reviewed by at least two authors, and studies were included in the meta-analysis based on a consensus decision (see Figure 1).

To meet the inclusion criteria, the studies had to (a) report group comparisons between postmenopausal women who received HT and matched controls, (b) employ fMRI, (c) report their results in a standard stereotactic space (either Talairach or Montreal Neurological Institute space), and (d) investigate working memory or working memory-related tasks. Studies that did not fulfill these requirements were excluded. Treatment, gender, and age were not restricted and the HT brain activation studies did not include a control.

The data extracted from each study included: a description of (a) each memory task and (b) foci of task-related activation changes in which the active and control states were different between the HT group and the control as well as (c) clinical data concerning hormone dose, years of estrogen use, and the age-matching of the control group. This study examined all foci that were reported as significant based on the above criteria designated in the included studies.

### ALE meta-analysis procedure

Two separate ALE meta-analyses were performed: one on fMRI studies reporting that brain activation was increased in postmenopausal women receiving HT compared to controls and a second on studies reporting that brain activation was decreased.

The meta-analyses were performed using GingerALE software, version 2.1.1 (Laird et al., 2005)^1^. The main idea behind ALE is to treat the reported foci as centers for 3D Gaussian probability distributions to capture the spatial uncertainty associated with each focus. Foci reported for any given study were then combined to a modeled activation (MA) map. The reported foci in the original studies in MNI space were transformed into Talairach space using the tal2icbm algorithm. All ALE data processing was performed using Brain Map Search and View software^2^. Statistical significance was assessed using an analytical equation. The assumption is reasonable for data imaging, based on an assumption of positive dependence (Genovese et al., 2002), and the false discovery rate (FDR) was set at 0.05 to correct the *p*-threshold for multiple comparisons (Laird et al., 2005). Each ALE map was overlaid onto an anatomical template generated by spatially normalizing the International Consortium for Brain Mapping (ICBM) template to the Talairach space (Kochunov et al., 2002). The resulting regions were anatomically labeled by reference to probabilistic cytoarchitectonic maps of the human brain.

## Results

### Number of studies found

The literature contained eight studies in which the effect of HT was investigated in working memory. In total, the meta-analysis was conducted on 103 postmenopausal women taking HT and 109 controls (see Table 1). These studies include all relevant treatment studies through 2014. Studies were excluded if they did not perform working memory tasks, or included a combination of estrogen and testosterone, or did not include a control group, or included women suffering from cognitive impairment.

**Table 1:** **Publications included in the meta-analysis, the tasks they employed, the number of subjects who were investigated, and the mean age of the women and their estrogen dose for the ALE meta-analysis**.

Study	Women in HT group/control	Field strength (T)	Task	Mean age (SD)	Years of estrogen use	Estrogen dose
Dumas et al. (2010)	10/10	3	Verbal *N*-back sequential letter	59.1 (5.5)	7.0 (6.8)	1 mg oral 17-β estradiol
Dumas et al. (2012)	12/12	3	Verbal *N*-back sequential letter	59.1 (5.5)	7.01 (6.8)	1 mg oral 17-β estradiol
Smith et al. (2006)	5/5	3	Visual working memory	56.9 (1.4)	0.17	5 μg ethinyl estradiol and 1 mg norethindrone acetate
Stevens et al. (2005)	8/8	1.5	Visual oddball	76.9 (3.9)	0.5	Ultralow dose estrogen (0.25 mg/day)
Persad et al. (2009)	5/5	3	Verbal memory	No mention	0.17	5 ugethinyl estradiol and 1 mg norethindrone acetate
Maki et al. (2011)	17/17	3	Verbal memory	60.16 (2.88)	>2	–
Joffe et al. (2006)	26/24	1.5	Verbal memory	50.0 (3.4)	0.25	17A-estradiol 0.05 mg/day patch
Berent-Spillson et al. (2010)	20/28	3	Visual working memory	64.4 (4.8)	13.3	Estrogens 0.625 mg/day

### Changes in neural activation to working memory tasks

**Figure 2:**
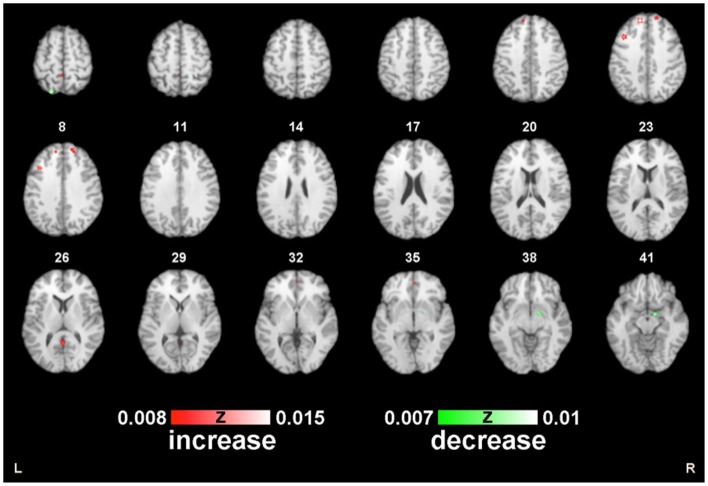
**ALE map of the postmenopausal women during working memory**. Decreased (green) and increased (red) activity following HT compared with the controls (*p* < 0.01; FDR-corrected; *k* > 100).

We performed two ALE analyses to investigate and aggregate the known data that exist regarding the effects of HT on neural activity in postmenopausal women during working memory tasks. The first analysis pooled the results of an increase in neural activation (8 experiments, 73 foci), and the second analysis pooled the results of a decrease in brain activation (3 experiments, 18 foci) (*p* ≤ 0.05 FDR-corrected; see Table 2; Figure 2).

**Table 2:** **Results from the ALE meta-analysis of treatment effects on studies in postmenopausal women (*p* < 0.05, FDR-corrected)**.

Location	BA	Peak activation			Volume (mm^3^)	ALE value × 10^−3^
		*x*	*y*	*z*		
**(A) AREAS OF INCREASED ACTIVATION FOLLOWING HT**						
Left cerebrum, frontal lobe, superior frontal gyrus	8	−10	46	36	416	14.50
Middle frontal gyrus	9	−38	18	34	368	13.08
Right cerebrum, superior frontal gyrus	9	16	50	34	256	10.67
Anterior lobe, culmen	–	0	−52	6	200	10.10
Parietal lobe, precuneus	7	0	−46	54	144	9.93
Right cerebrum, anterior cingulate	32	4	46	−2	112	9.86
Paracentral lobule	5	−2	−44	58	–	9.78
**(B) AREAS OF DECREASED ACTIVATION FOLLOWING HT**						
Right cerebrum, limbic lobe, parahippocampal gyrus	28	14	−6	−12	296	9.93
Left cerebrum, parietal lobe, superior parietal lobule	7	−18	−72	57	280	10.36

*Results are shown in Talairach coordinates. Significance threshold is *p* < FDR 0.01 and *k* > 100*.

**Figure 3:**
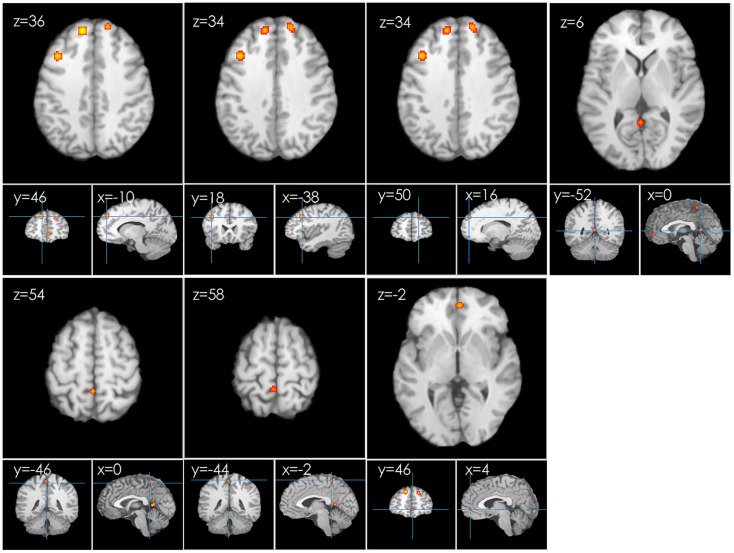
**An ALE map presenting the increased activity in postmenopausal women undergoing HT during working memory tasks compared with the controls (*p* < 0.01; FDR-corrected; *k* > 100)**.

In the first meta-analysis, five clusters of increased task-related activity after HT were found in the left frontal lobe, superior frontal gyrus (BA 8), middle frontal gyrus (BA 9), parietal lobe, precuneus (BA 7), paracentral lobule (BA 5), limbic lobe, and anterior cingulate (BA 32) (*p* < 0.01 FDR-corrected, *k* > 100; see Figure 3).

**Figure 4:**
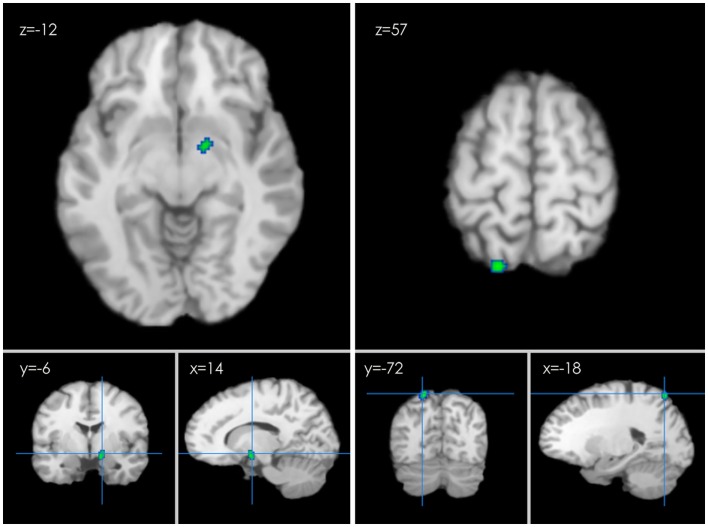
**An ALE map presenting the decreased activity in postmenopausal women undergoing HT during working memory tasks compared with the controls (*p* < 0.01; FDR-corrected; *k* > 100)**.

Several clusters of decreased activation after HT were detected in the second ALE analysis. Clusters of decreased activation were located in the right limbic lobe, parahippocampal gyrus (BA 28), left parietal lobe, and superior parietal lobule (BA 7) (*p* ≤ 0.05 FDR-corrected; see Table 2; Figure 4).

### HT and performance of working memory

Four papers measured the relationship between HT and performance on verbal memory task. Two studies indicated that HT use increases frontal lobe activity during verbal memory tasks, but the degree of this increase is related to cognitive load (Dumas et al., 2010). One study demonstrated that HT results in changes in neural activation in verbal memory circuits in postmenopausal women and suggests that estrogen may enhance the overall efficiency of verbal memory processes in postmenopausal women (Persad et al., 2009). Another study suggested that HT selectively enhances verbal perseveration and is an important component of executive function by increasing activation in the inferior frontal, dorsolateral prefrontal, and posterior parietal regions (Joffe et al., 2006). One study indicated that no significant differences in visual task performance were found in response to HT. However, there was evidence that the HT group had both reductions and increases in the amplitude of hemodynamic response in some regions, including the occipital and parietal lobes, motor cortex, anterior cingulate, and PFC (Stevens et al., 2005).

### Sub-analyses for estrogen long-term use and short-term use

In further sub-analysis, we investigated the effects of estrogen experience. The brain network supporting long experience [usually more than 2 years (Barrett-Connor and Stuenkel, 2001)] estrogen use (27 Foci, 4 Experiments) included cluster of activity in the left limbic lobe, right frontal lobe, and left anterior lobe. Short experience [usually <2 years (Barrett-Connor and Stuenkel, 2001)] included clusters of activity in the right front lobe, left front lobe, limbic lobe, and parietal lobe. We get similar behavioral outcomes after excluding short-term estrogen use. This suggests that long-term hormone use have a persisting chronic effect in postmenopausal women.

## Discussion

Historically, cognitive benefits of postmenopausal hormone therapy (HT) have not been consistently demonstrated and the brain activation patterns are inconsistent. In this study, we addressed the cognitive effects of hormones in women and performed an ALE meta-analysis on functional brain imaging studies to investigate and aggregate the known data that exist regarding the effects of HT on neural activity in postmenopausal women during working memory tasks. Our results indicate that hormone use is associated with increased regional brain activation during working memory task, with women in hormone-treatment groups exhibiting a more robust neural response than placebo-treated women. Women receiving HT exhibited increases in brain activation in the frontal lobe and the anterior cingulate cortex (ACC) (see Table 2; Figure 3), a region where decreased activity can be predictive of Alzheimer’s disease (Rasgon et al., 2005). This finding suggests that HT may enhance brain function. The frontal lobe and the ACC may be important for processing lexical information and for the associated cognition and decision-making (Bush et al., 2000). This supports our prior hypothesis that HT maintains a positive effect on working memory processes.

Activation in the frontal lobes has been identified in postmenopausal women after estradiol treatment (Dumas et al., 2010). The frontal lobes are of paramount significance in determining emotions and judgments related to sympathy, which is defined as the ability to perform daily activities, personality manifestations, and decisions (Badre et al., 2009; Tiemeier et al., 2010). The hormone effects on working memory tasks may occur through an effect on the frontal lobe. Activation of frontal regions during memory tasks has been associated with the active maintenance of information (Cohen et al., 1997). Activation in frontal regions during a working memory task has been related to improved performance in older subjects (Davis et al., 2008). Hormones exert a greater modulatory effect on the function of the frontal lobe when working memory load is increased (Dumas et al., 2010), and episodic memory tasks are associated with the frontal lobe (Tulving et al., 1994). fMRI–BOLD signal in the frontal lobes, a region central to memory processing, positively correlated to task accuracy, implying a cognitive benefit to the increased activity measured during the memory task (Joffe et al., 2006; Dumas et al., 2010).

Similarly, across our ALE meta-analyses, we found that, after HT, postmenopausal women exhibited greater activity in the ACC than controls. The ACC plays an important role in integrating cognitive and emotional processes to support goal-directed behavior (Krause-Utz et al., 2014). On the other hand, ACC is involved in rational cognitive functions, including reward anticipation, impulse control decision-making, empathy (Bush et al., 2000), and emotion (Decety and Jackson, 2004; Jackson et al., 2006). This finding in the present study is consistent with the effect of HT on the emotion-processing circuitry in postmenopausal women (Frey et al., 2010; Shafir et al., 2012). However, another study revealed contradictory results in which the BOLD responses were reduced in the dorsolateral PFC and the dorsal anterior cingulate of the HT group compared with the control group during a negative emotion task. These differences between the HT and control groups may reflect a recovery of emotional responsiveness in these older women following HT (Love et al., 2010).

Additionally, decreased activation in the parahippocampal gyrus (BA 28), including left parietal lobe and superior parietal lobule (BA 7) (see Table 2; Figure 4), was observed in the HT group. Importantly, these regions may also support cognitive functions, including verbal memory (Zec and Trivedi, 2002; Sherwin, 2012). The parahippocampal gyrus is a gray matter cortical region of the brain that surrounds the hippocampus. The key role of this region is memory encoding and retrieval. Abnormalities may indicate underlying conditions, such as schizophrenia, Alzheimer’s disease, and hippocampal sclerosis (Ferreira et al., 2003). The observation of hyper-activation in the parahippocampal gyrus is consistent with the finding of Dr Maki leading investigators that women receiving HT exhibit decreased regional cerebral blood flow in the right precuneus, the dorsal frontal gyrus, and the parahippocampal gyrus during a verbal memory task (Resnick et al., 1998). Their later study suggested that deactivation in the left parahippocampal gyrus relates to better verbal memory performance, and this effect was specific to the verbal recognition condition (Maki et al., 2011). Another study found sustained decreases in activation in bilateral regions of parahippocampal cortex, suggesting that the parahippocampal gyrus might become deactivated when participants entered into a sustained state of retrieval during recognition tasks (Donaldson et al., 2001). Together, the findings of decreased parahippocampal activation in perimenopausal HT users suggest that perimenopausal HT enhances both state-dependent and recollective processes contributing to verbal recognition performance.

Sub-analyses for estrogen long-term and short-term use revealed almost the same results to the main results of HT group. The goal of this study was to evaluate differences in working memory after hormone use compared to placebo use, so women who recently quit long-term hormones were included along with current hormone users. Apart from a statistically significant difference in mean years of education, these two groups were demographically similar (although the smaller sample size used to compare current and past users may increase the likelihood of a type II error). After accounting for variations in age, education, and age at hormone initiation, long-term users did not have more activation than short-tem users in any part of the brain. Long-term users showed slightly more activation than short-term users in only one small region within the prefrontal cortex. While it is difficult to make conclusions based on one differing region, these results may be consistent with others that have indicated a persisting cognitive benefit to past hormone users (Hogervorst et al., 2009). Other than this region, the activation patterns between these two HT groups were sufficiently similar to justify including them in the same groups when comparing hormone ever-users to placebo-users.

This study has some limitations. First, methodological limitations led to a small number of available studies, and the sample sizes are small. Second, trials recruit women who may not be representative of the general population. Third, differences among the studies in the education, general health, motivation level, and age. These differences raise the possibility that the controls and HT groups may differ with respect to characteristics that could influence brain function. We consider this limitation unavoidable. Third, many trials are too short to evaluate long-term effect of HT. However, such trials provide more highly reliable evidence of the effectiveness of a treatment than observational studies (Miller and Kearney, 2004). Fourth, the functional imaging studies included employed different working memory tasks and the years of estrogen use are various, which could result in a source of heterogeneity. Fifth, the estrogen dose of HT was not identical across the included studies, which may influence the effect of estrogen on postmenopausal women.

## Conclusion

In summary, the present study demonstrated that women who received HT exhibited increases in brain activation in the frontal lobe and ACC. In addition, decreased activation in the parahippocampal gyrus (BA 28), left parietal lobe, and superior parietal lobule (BA 7) were detected. The results are consistent with our hypothesis. Together, the findings of enhanced frontal lobe activation and decreased parahippocampal activation in perimenopausal HT users suggest that HT enhances both state-dependent and recollective processes contributing to cognitive performance.

## Author Contributions

KL designed the study and wrote the protocol. XH and YH managed the literature searches and undertook the statistical analysis, and KL, XH, LY, JL, and DZ wrote the manuscript. JZ and YL took part in the modification and further analysis of the final manuscript. All authors contributed to and have approved the final manuscript.

## Conflict of Interest Statement

The authors declare that the research was conducted in the absence of any commercial or financial relationships that could be construed as a potential conflict of interest.

## Footnotes

^1^http://brainmap.org/ale

^2^http://brainmap.org
